# Periocular Sebaceous Carcinoma: A Rare Entity in Mexico

**DOI:** 10.7759/cureus.116780

**Published:** 2026-09-23

**Authors:** Bryan Salvador Sol, Teresa Jannete Ortega Valls, Amara Hazel Solorio Rivera, Brenda Ayuzo Del Valle, Diana Maria Ibarra Tostado, Víctor Manuel Tarango-Martinez

**Affiliations:** 1 Dermatology, Instituto Dermatológico de Jalisco "Dr. José Barba Rubio", Zapopan, MEX

**Keywords:** dermoscopy, meibomian gland, mohs micrographic surgery, muir-torre syndrome, periocular sebaceous carcinoma

## Abstract

Sebaceous carcinoma (SC) is a malignant neoplasm arising from the sebaceous glands. Its periocular location, originating from the meibomian or Zeis glands, can clinically mimic benign lesions such as chalazion or chronic blepharitis, which may delay diagnosis.

We present a case of an elderly male patient with a history of nodular basal cell carcinoma, type 2 diabetes mellitus, and systemic arterial hypertension, who presented to the dermatology clinic with an asymmetric neoformation on the right lower eyelid, reddish in color with whitish-yellow areas and ulceration with a hematic crust. Dermoscopy showed polymorphic vessels and structureless yellowish areas. Histopathological examination revealed a malignant epithelial tumor with clear-cytoplasm cells, pleomorphic nuclei, and focal necrosis, with sebaceous differentiation confirmed by immunohistochemistry positive for epithelial membrane antigen (EMA). Surgical resection with a wide safety margin and reconstruction using a Mustardé flap were performed, achieving tumor-free histological margins. Clinical and imaging follow-up showed no evidence of recurrence or metastasis.

In this patient, wide local excision with complete histopathological evaluation confirmed complete removal of the lesion with clear surgical margins, with no evidence of recurrence during follow-up.

## Introduction

Sebaceous carcinoma (SC) is an uncommon malignant neoplasm derived from the sebaceous glands, recognized for its aggressive behavior and high rate of recurrence and metastasis. SC has traditionally been classified into periocular and extraocular forms, with the periocular form historically reported to account for approximately 75% of cases [1]. In Western populations, periocular SC accounts for 1% to 5.5% of malignant eyelid tumors, with an estimated incidence of 0.1 to 0.23 per 100,000 person-years in countries such as the United States and the Netherlands [2-4]. This tumor predominantly affects adults over 60 years of age, with a slight female predominance in periocular cases [4,5]. Although rare, it can occur in younger patients, and its presentation in individuals under 40 years of age should raise suspicion of an association with Muir-Torre syndrome, a variant of Lynch syndrome involving microsatellite instability and mutations in DNA mismatch repair genes (MLH1, MSH2, MSH6) [1,6]. Despite its clinical relevance given its behavior, reports in Hispanic patients remain limited; we therefore present the following clinical case with the aim of highlighting its diagnostic and therapeutic challenges.

## Case presentation

An 87-year-old male patient, with a history of nodular basal cell carcinoma, type 2 diabetes mellitus, and systemic arterial hypertension, presented to the dermatology outpatient clinic with an eight-month history of a periocular lesion associated with bleeding and abrupt growth over the preceding three months. Physical examination revealed a dermatosis of the right periocular region, on the lower eyelid at the level of the superior zygomatic border, consisting of a neoformation measuring 1.2 × 1.0 × 0.3 cm, asymmetric, reddish in color, with whitish-yellow areas and a lateral ulcer covered by a hematic crust (Figure 1). Polarized-light dermoscopy showed polymorphic vessels and structureless whitish-yellow areas (Figure 2).

**Figure 1:**
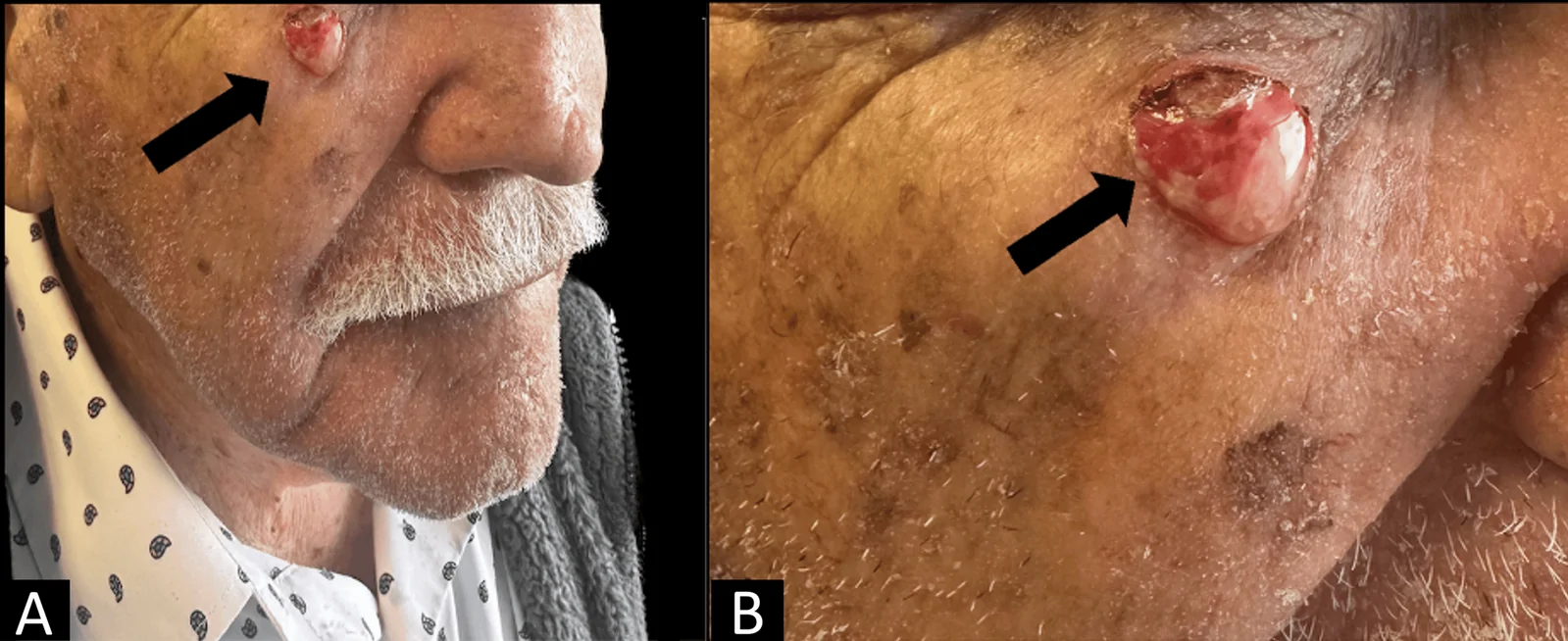
(A, B) Clinical presentation showing a 1.2 × 1.0 × 0.3 cm asymmetric erythematous neoplasm with whitish-yellow areas and a lateral ulceration with a hemorrhagic crust on the surface (black arrows).

**Figure 2:**
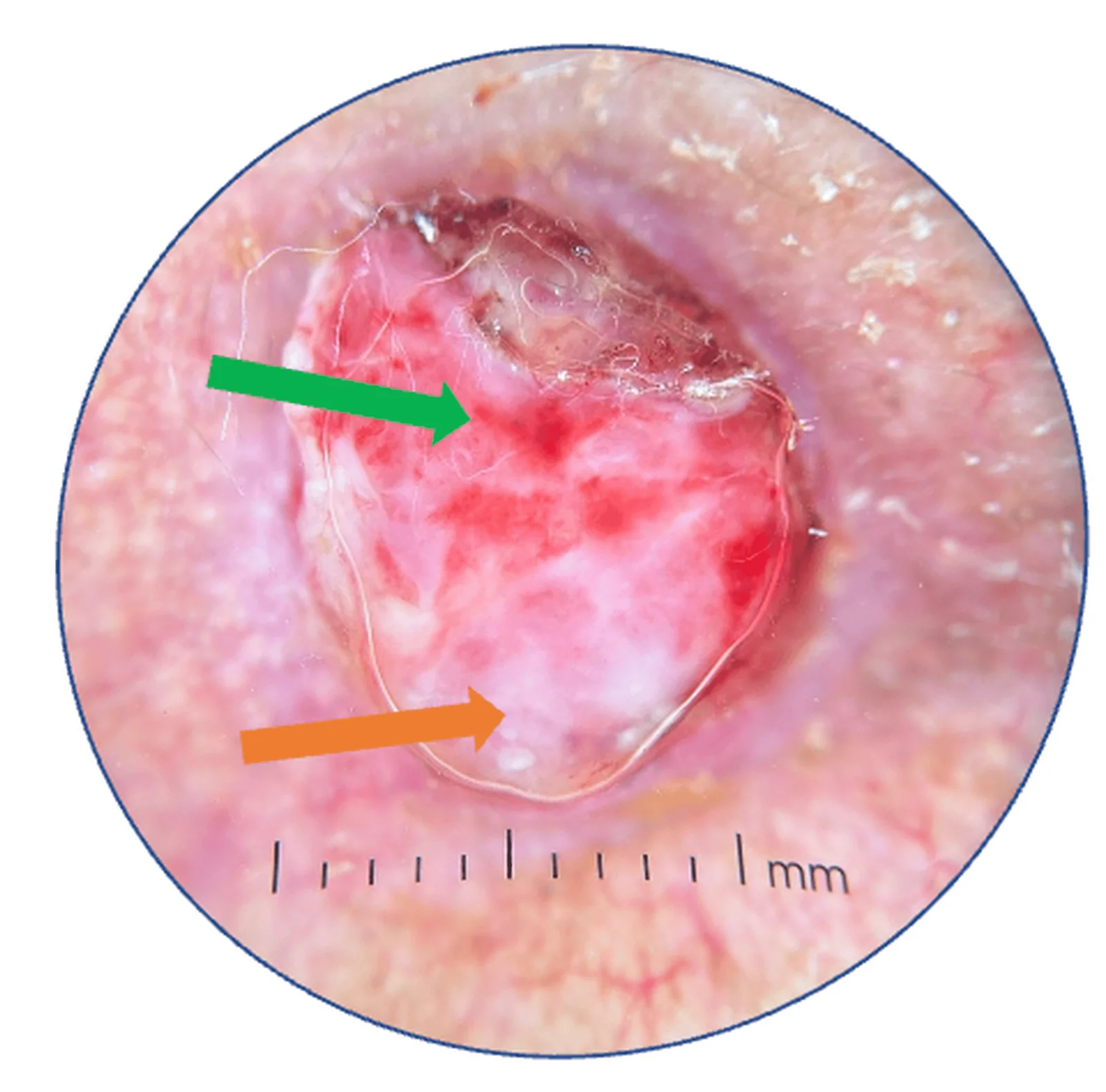
Polarized light dermoscopy revealing polymorphous vessels (green arrow) and structureless whitish-yellow areas (orange arrow).

An excisional biopsy was performed; histopathological examination showed an ulcerated epidermis and a malignant epithelial tumor arranged in nests, composed of cells with clear cytoplasm, pleomorphic nuclei, and areas of tumor necrosis (Figure 3A). These findings led to a diagnosis of a moderately differentiated epithelial tumor, confirmed by immunohistochemistry with positive staining for epithelial membrane antigen (EMA ++) (Figure 3B). Histopathology revealed classic features of SC: cells with vacuolated clear cytoplasm, pleomorphic nuclei, and focal central necrosis [2,7]. EMA positivity reinforced sebaceous differentiation, a reliable marker in the differential diagnosis of malignant adnexal tumors.

**Figure 3:**
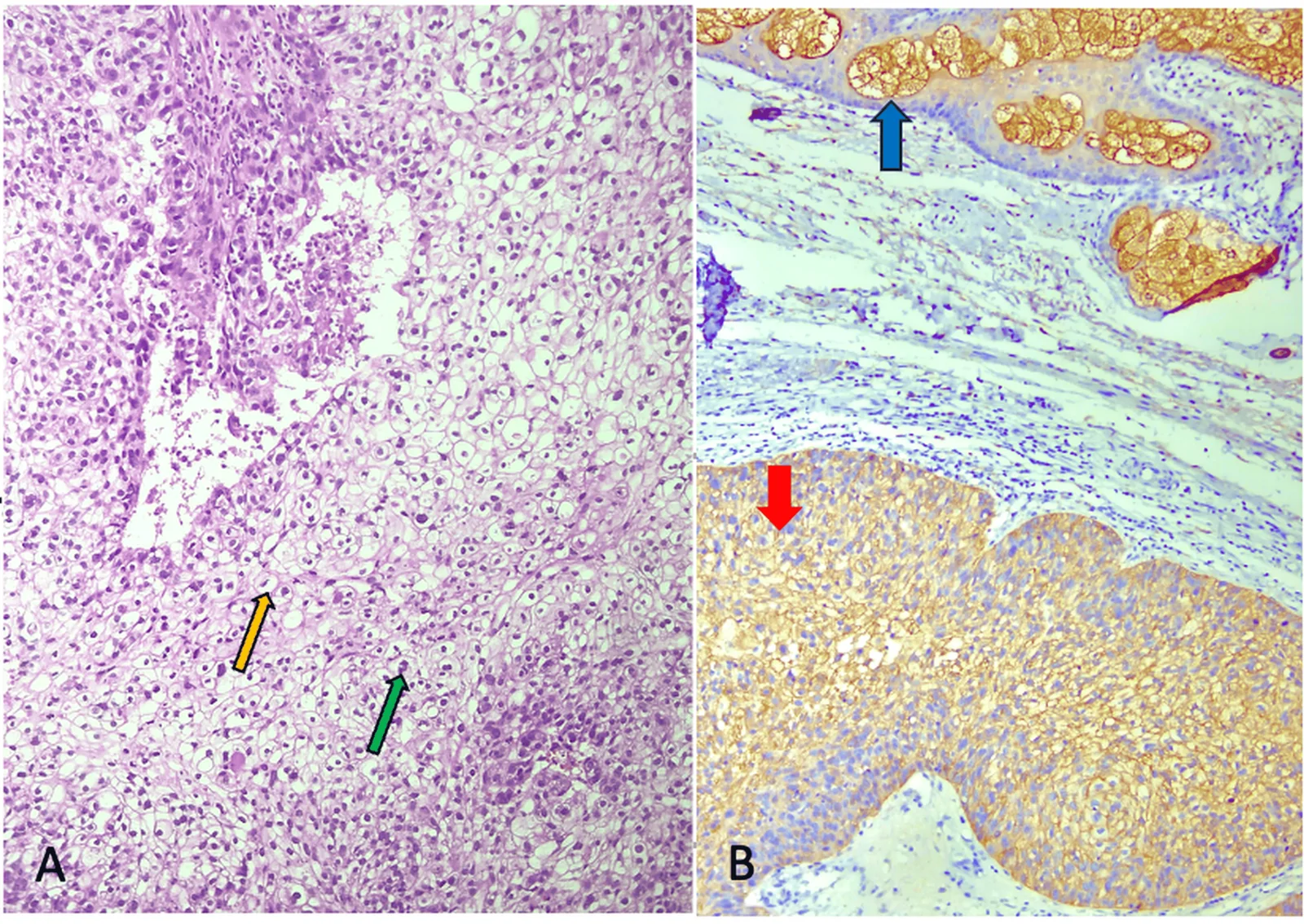
(A) Hematoxylin and eosin (H&E) staining showing epithelial cells arranged in clusters, with cells exhibiting clear cytoplasm (yellow arrow) and pleomorphic nuclei (green arrow). (B) Immunohistochemistry showing epithelial membrane antigen (EMA) positivity in the tumor (red arrow) and in the sebaceous gland, serving as an internal positive control (blue arrow).

The patient underwent surgical resection with wide margins, using a 1.2 cm safety margin. Closure was performed with a Mustardé cheek rotation flap (Figure 4).

**Figure 4:**
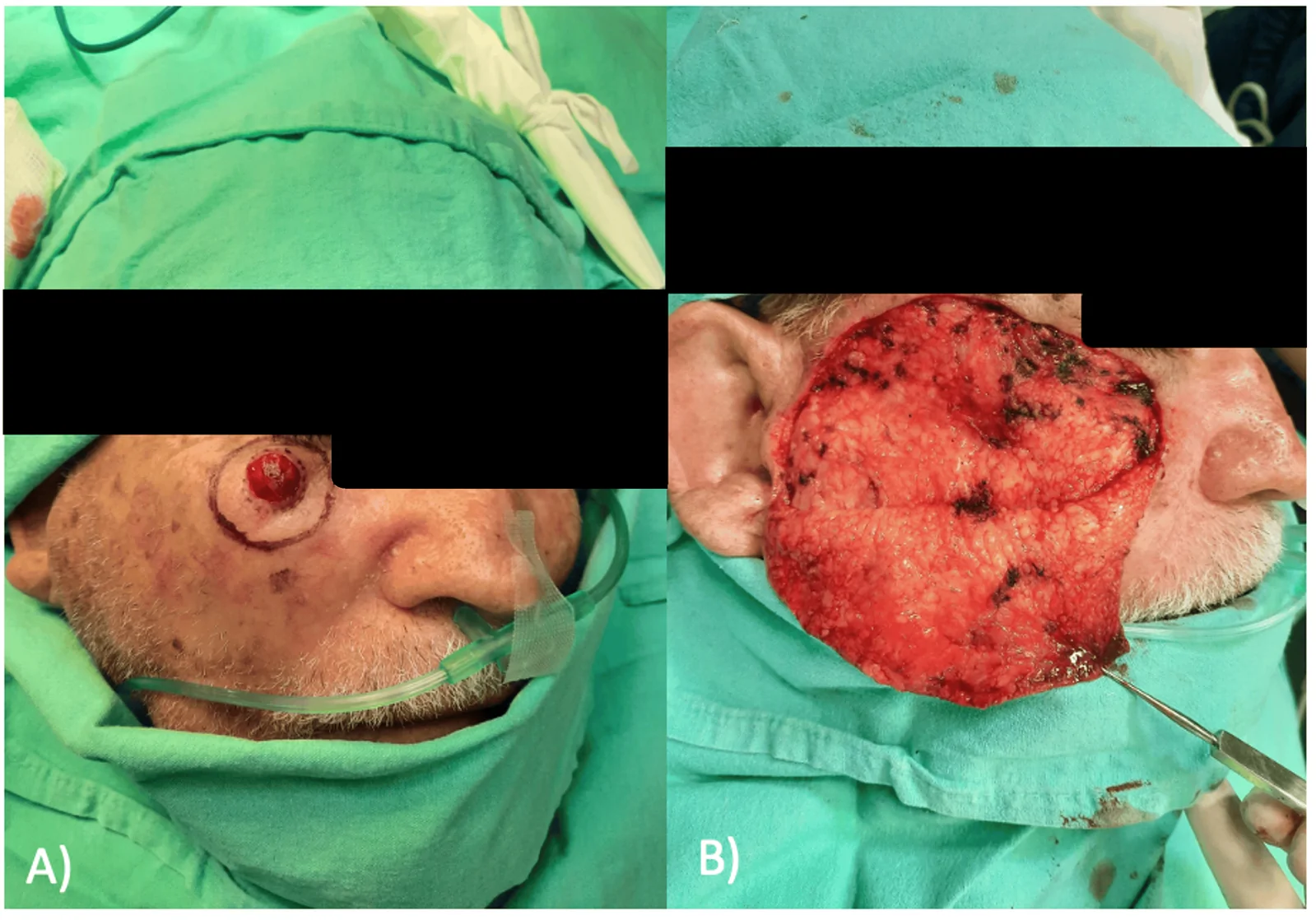
Surgical procedure depicting surgical margin of 1.2 cm (A) and Mustardé flap (B).

Histopathological analysis of the surgical specimen reported tumor-free margins. There was no involvement of the eyelid margin or tarsal plate. Based on the American Joint Committee on Cancer (AJCC) 8th edition staging system for eyelid carcinoma, the 12 mm lesion was classified as T2a [8]. No regional lymphadenopathy or distant metastatic disease was identified, corresponding to N0M0 and clinical stage IB. Postoperative follow-up visits showed a well-approximated linear scar (Figure 5). At three months, computed tomography showed no evidence of recurrence or metastatic disease. The patient remains under close clinical follow-up two years after wide surgical resection, with no evidence of tumor progression.

**Figure 5:**
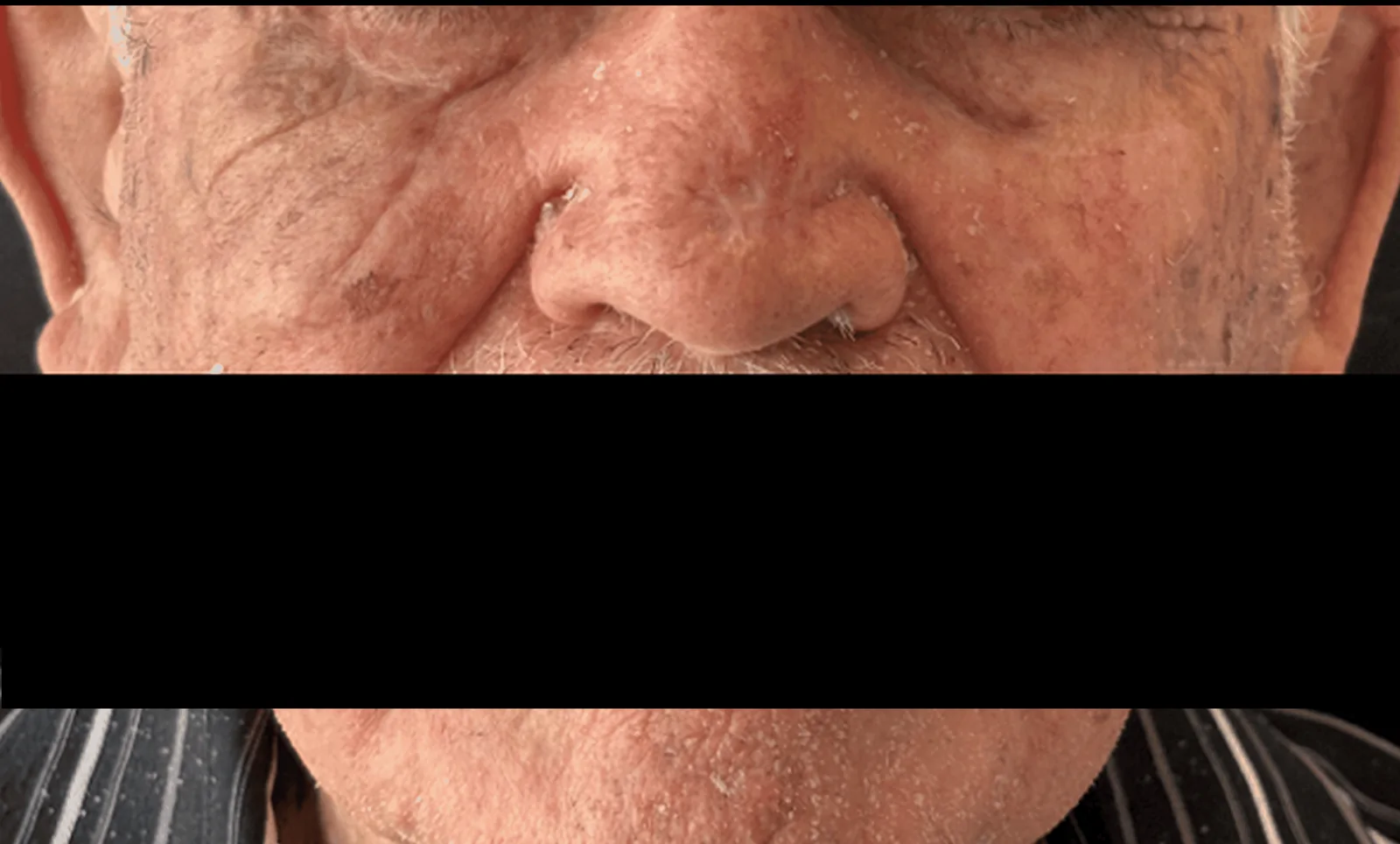
Three-month postoperative follow-up photograph showing an imperceptible scar and no recurrence.

## Discussion

Historically, sebaceous gland tumors - particularly those located on the eyelid - generated considerable debate regarding their existence, behavior, and classification. In 1956, Straatsma was the first to systematically distinguish between benign adenomas and true carcinomas of the meibomian glands, describing their clinical, histological, and prognostic features [9].

Periocular SC arises from the meibomian, Zeis, or caruncular glands and has a marked tendency toward local spread, pagetoid invasion, and regional metastasis, primarily to the preauricular and parotid lymph nodes [10]. Clinically, it may present as a firm, painless, slow-growing yellowish or erythematous nodule, or as diffuse eyelid thickening; its resemblance to benign lesions delays diagnosis by an average of one to two years from symptom onset [4,7,11].

Dermoscopy has emerged as a valuable diagnostic tool. Common findings include whitish-pink areas, yellow structures, polymorphic vessels, and structureless yellow areas [11]. These features help distinguish SC from other neoplasms such as squamous cell carcinoma or basal cell carcinoma, although histopathological confirmation remains necessary. Despite the development of complementary tools such as immunohistochemistry - reserved for poorly differentiated cases - diagnosis continues to rely on conventional histopathological evaluation, which identifies key features such as lobular pattern, vacuolated-cytoplasm cells, and nuclear atypia.

Regarding treatment, complete surgical resection remains the primary therapeutic strategy, with Mohs micrographic surgery considered the first-line treatment due to its ability to assess margin control while preserving healthy tissue [1]. This technique has been shown to significantly reduce local recurrence rates compared with conventional wide excision, particularly in anatomically sensitive locations such as the eyelid [1]. Although Mohs micrographic surgery is regarded as the gold standard - given its ability to assess intraoperative margins and reduce recurrence rates (6.8% vs. 23.4% with conventional excision) [6] - in our clinical setting, wide local excision with a 1.2 cm clinical margin was chosen, a practice also supported by guidelines such as the German S1 guideline, which recommends margins of 5 to 10 mm in the absence of Mohs surgery [1]. In this case, complete histological control was achieved, with adequate cosmetic and functional outcomes owing to reconstructive planning using a Mustardé rotational flap.

It is well recognized that this entity can mimic common benign lesions such as chalazion, chronic blepharitis, or basal cell tumors, which contributes to diagnostic delay [2,7].

## Conclusions

This case underscores the importance of maintaining a high index of suspicion for eyelid lesions with rapid or abrupt progression, particularly in elderly patients, in whom SC can mimic benign entities such as chalazion or chronic blepharitis. Dermoscopy is a valuable tool in initial clinical evaluation, revealing vascular patterns and specific structures suggestive of a malignant adnexal neoplasm. Likewise, the need for structured follow-up is emphasized, including periodic clinical examination and imaging studies to rule out local recurrence or regional and visceral metastasis. Our patient remains free of recurrence or dissemination to date, confirming the efficacy of the surgical approach performed. This reinforces that, in settings where Mohs micrographic surgery is unavailable, wide local excision with adequate margins and complete histological evaluation constitutes a valid, safe, and optimal therapeutic alternative.
